# Impaired Relaxation in Aorta from Streptozotocin-diabetic Rats: Effect of Aminoguanidine (AMNG) Treatment

**DOI:** 10.1155/EDR.2000.145

**Published:** 2000

**Authors:** Sibel Özyazgan, Yesim Unlucerci, Selda Bekpinar, Ahmet Gökhan Akkan

**Affiliations:** 1 University of Istanbul Cerrahpasa Faculty of Medicine Department of Pharmacology Istanbul 34303 Turkey

## Abstract

*Aim* The effect of 8 weeks′ streptozotocin (STZ)- induced diabetes and aminoguanidine (AMNG), the inhibitor of advanced glycosylation reaction, treatment on arteriolar reactivity to vasoactive substances was investigated *in vitro*.

*Materials and Methods* Studies were performed in untreated control rats (*n* = 10), STZ-induced (60 mg/kg i.v.) diabetic rats (*n* = 10), AMNG-treated (600 mg/l given in drinking water throughout 8 weeks) control rats (*n* = 10) and AMNG-treated (600 mg/l given in drinking water, beginning at 72h after STZ and throughout 8 weeks of diabetes) diabetic rats (*n* = 10). Results are expressed as the mean ±s.e. Relaxant responses are expressed as a percentage (%) relaxation of noradrenaline-induced tone. Statistical comparisons were made by one-way analysis of variance (ANOVA) followed by Tukey–Kramer multiple comparisons test.

*Results* 1. The decreased body weights (205 ± 6 g) and increased blood glucose levels (583 ± 8 mg/dl) of diabetic rats were partially restored by treatment of aminoguanidine (253 ± 6 g, *p* < 0.05 and 480 ± 14 mg/dl, *p* < 0.001, respectively). 2. Diabetes caused a 71% deficit in maximal endothelium-dependent relaxation to acetylcholine for noradrenaline precontracted aortas (*p* < 0.001). AMNG treatment prevented the diabetes-induced impairment in endothelium dependent relaxation (58 ± 8%) to acetylcholine, maximum relaxation remaining in the non-diabetic range (78 ± 4%). 3. Neither diabetes nor treatment affected endothelium-independent relaxation (pD_2_ and max. Relax.) to sodium nitroprusside. 4. Vasoconstrictor responses (pD_2_ and Max. Contraction) to noradrenaline and KCl were not influenced by the diabetic state and treatment.

*Conclusion* Our data suggest that 8 weeks of experimental diabetes is associated with a decreased endothelium-dependent vasodilatation. AMNG treatment may prevent diabetes-induced endothelial dysfunction. This may be mediated *via* the prevention of advanced glycosylation end product formation, the enhanced release of vasodilator substances such as prostacyclin, the increased elasticity of blood vessels, the antioxidant activity and inhibitor activity of enzyme aldose-reductase by AMNG.


*Int. Jnl. Experimental Diab. Res.*, Vol. 1, pp. 145–153
Reprints available directly from the publisher
Photocopying permitted by license only

© 2000 OPA (Overseas Publishers Association) N.V.
Published by license under
the Harwood Academic Publishers imprint,
part of The Gordon and Breach Publishing Group.
Printed in Malaysia.

### Impaired Relaxation in Aorta from Streptozotocin-diabetic Rats: Effect of Aminoguanidine (AMNG) Treatment

SIBEL ÖZYAZGAN, YESIM UNLUCERCI, SELDA BEKPINAR and AHMET GÖKHAN AKKAN*

*University of Istanbul, Cerrahpasa Faculty of Medicine, Department of Pharmacology, 34303, Istanbul, Turkey*

*(Received 27 September 1999; In final form 10 February 2000)*

***Aim*** **The effect of 8 weeks' streptozotocin (STZ)-induced diabetes and aminoguanidine (AMNG), the inhibitor of advanced glycosylation reaction, treatment on arteriolar reactivity to vasoactive substances was investigated *in vitro*.**

***Materials and Methods*** **Studies were performed in untreated control rats (*n*=10), STZ-induced (60 mg/kg i.v.) diabetic rats (*n*=10), AMNG-treated (600 mg/l given in drinking water throughout 8 weeks) control rats (*n*=10) and AMNG-treated (600 mg/l given in drinking water, beginning at 72 h after STZ and throughout 8 weeks of diabetes) diabetic rats (*n*=10). Results are expressed as the mean ± s.e. Relaxant responses are expressed as a percentage (%) relaxation of noradrenaline-induced tone. Statistical comparisons were made by one-way analysis of variance (ANOVA) followed by Tukey–Kramer multiple comparisons test.**

***Results*** **1. The decreased body weights (205 ± 6 g) and increased blood glucose levels (583 ± 8 mg/dl) of diabetic rats were partially restored by treatment of aminoguanidine (253 ± 6 g, $p < 0.05$ and 480 ± 14 mg/dl, $p < 0.001$, respectively). 2. Diabetes caused a 71% deficit in maximal endothelium-dependent relaxation to acetylcholine for noradrenaline precontracted aortas ($p < 0.001$). AMNG treatment prevented the diabetes-induced impairment in endothelium dependent relaxation (58 ± 8%) to acetylcholine, maximum relaxation remaining in the non-diabetic range (78 ± 4%). 3. Neither diabetes nor treatment affected endothelium-independent relaxation ($pD_2$ and max. Relax.) to sodium nitroprusside. 4. Vasoconstrictor responses ($pD_2$ and Max. Contraction) to noradrenaline and KCl were not influenced by the diabetic state and treatment.**

***Conclusion*** **Our data suggest that 8 weeks of experimental diabetes is associated with a decreased endothelium-dependent vasodilatation. AMNG treatment may prevent diabetes-induced endothelial dysfunction. This may be mediated *via* the prevention of advanced glycosylation end product formation, the enhanced release of vasodilator substances such as prostacyclin, the increased elasticity of blood vessels, the antioxidant activity and inhibitor activity of enzyme aldose-reductase by AMNG.**

*Keywords*: Diabetes, aorta, streptozotocin, aminoguanidine, endothelium

### INTRODUCTION

Hyperglycaemia is the primary cause of diabetic micro and macrovascular complications.[1,2] Several major mechanisms have been proposed for hyperglycaemia-induced tissue damage, including advanced glycation end product

*Corresponding author. Tel.: +90 212 586 15 52, Fax: +90 212 633 01 31, e-mail: gakkan@istanbul.edu.tr

(AGE) formation, altered intracellular redox state, increased polyol pathway flux, apoptosis and increased de novo diacylglycerol synthesis with resultant activation of protein kinase C isoform. [3–7]

Studies on vascular reactivity using the STZ-diabetic rat have reported inconsistent results with increased, decreased and unchanged responsiveness to noradrenaline, KCl, acetylcholine and sodium nitroprusside. [10–14]

Today, in addition to the prevention of hyperglycaemia by use of insulin and oral hypoglycaemic agents, it has been also used and tested some agent such as aminoguanidine for the prevention and treatment of complications in Diabetes Mellitus. Aminoguanidine is a small, hydrazine compound structurally identical to the amino-terminal group of arginine and several laboratories have reported beneficial effects of aminoguanidine in some diabetic complications. The mechanisms for these effects of AMNG were accompanied by reduction of advanced glycation end product (AGE) formation, the inhibition of an isoform of nitric oxide synthase (iNOS) and reactive oxygen species (ROS) formation, lipid peroxidation and oxidant-induced apoptosis. [15–18]

The present study was designed to define whether AMNG has beneficial effects on altered vascular reactivity of the thoracic aortas in streptozotocin-diabetic rats.

### MATERIALS AND METHODS

### Animals

Albino Wistar rats were bred in our laboratory and nourished ad libitum with standart pellet diet and had free access to tap water. Adult male and female Wistar rats ($n=40$), weighing 200–300 g, were divided into four groups:

(i) Normal Control (=NC) ($n=10$),

(ii) STZ-diabetic group (=DC) ($n=10$),

(iii) AMNG-treated control group (AG–C) ($n=10$),

(iv) AMNG-treated diabetic group (AG+D) ($n=10$).

### Induction of Experimental Diabetes

Rats were injected intravenously (into the lateral tail vein) with STZ (60 mg/kg)(Sigma, S-0130, Lot. 128H1045). STZ was freshly prepared in 0.25 ml saline. The rats were then maintained for 8 weeks with free access to food and water. Duration of the experimental period, blood glucose levels and body weights were measured beginning from 8th week.

### Aminoguanidine Treatment

The rats in group III (AMNG-treated control group) and group IV (AMNG-treated STZ-diabetic group) were given 600 mg/l AMNG in drinking water (in group AG + D, beginning at 72 h after STZ) for 8 weeks. The dosage in the drinking water was three times lower for the diabetic animals because their water intake is three times higher than that of nondiabetic animals. [45] In the dosage calculation, followed formula was used:

**[(mg AG in per ml drinking water**×ml daily water intake)]×**[(1000/body weight)].**

### Aortic Strips

Rats were sacrified by stunning followed by decapitation. A segment (3–5 cm) of thoracic aorta was removed and placed in an ice-cold Krebs–Ringer solution (KRS) of the following composition (mmol/L): NaCl (118), KCl (4.7), $CaCl_2$ (2.5), $MgSO_4 \cdot 7H_2O$ (1.2), $KH_2PO_4$ (1.2), $NaHCO_3$ and glucose (11.1) and then trimmed free of adhering fat and connective tissue and cut into rings of 3 mm width. The rings were opened by cutting the vessels longitudinally. Subsequently, they were fixed with stainless steel clips at both ends and then placed in 20 ml organ baths containing KRS, gassed with carbogen (95% $O_2$ + 5% $CO_2$) providing pH 7.4 at 37°C. The preparation were connected to isometric force displacement transducer (MAY, FDT 10-A isometric transducer) connected to MAY, TDA 97 polygraph and were equilibrated for 90 min at optimal resting tension of 2 g. During this time, the KRS in the organ bath was replaced every 20 min.

### Concentration-response Curves

After equilibration, the thoracic aorta strips were exposed to $10^{-5}$ mol/L ($\sim EC_{90}$) noradrenaline until the contraction reached the plateau (approximately 15 min) in order to measure the fast and slow components of vascular response to noradrenaline. The fast component was measured from the baseline to the point at which the rate of contraction started to decrease abruptly. The slow component was measured from this point to the top of the contraction. The total response was the sum of these two components.[16] Concentration-response curves were obtained with noradrenaline. Noradrenaline ($10^{-9}/3 \cdot 10^{-9} - 10^{-4}/3 \cdot 10^{-4}$ mol/L) was added in a cumulative manner until a maximal response was achieved. After the addition of each dose, a plateau response was obtained before the addition of a subsequent dose. Cumulative relaxation curves to acetylcholine (Ach) ($10^{-8} - 10^{-4}$ mol/L) and sodium nitroprusside (SNP) ($10^{-8} - 10^{4}$ mol/L) were obtained in each strip precontracted submaximally (approximately $EC_{90}$, $10^{-5}$ mol/L) by addition of noradrenaline. Concentration-response curves were obtained with KCl. KCl (20, 40, 60 and 80 mmol/L) was added in a cumulative manner until a maximal response was achieved. After the addition of each dose, a plateau response was obtained before the addition of a subsequent dose.

At the end of each experiment, tissue was blotted dry, measured and weighed.

### Data Analysis

Contractile responses to noradrenaline, KCl and relaxations to Ach (Inh.%) and SNP were calculated as the increase (and decrease for Ach and SNP) in tension (mg) in response to the agonist per mg of aorta. Agonist $pD_2$ value ($= -\log EC_{50}$) was calculated from each agonist concentration-response curve by linear regression analysis of the linear portion of the curve and taken as a measure of the sensitivity of the tissues to each agonist. All values are expressed as (mean ± SE). Statistical analysis of the data was performed using one-way analysis of variance (ANOVA) followed by Tukey–Kramer Multiple comparisons test. $p < 0.05$ was considered as statistically significant.

### RESULTS

### General Characteristics

Data showing changes in body weight and final blood glucose concentration for all groups are summarized in Table I.

The body weights of diabetic rats (205 ± 7 g) were significantly lower than NC ($p < 0.001$), AG − C ($p < 0.001$) and AG + D ($p < 0.05$) rats.

Eight weeks after injection, all rats treated with STZ exhibited severe hyperglycaemia and their blood glucose levels (583 ± 8 mg/dl) were significantly higher than those of NC ($p < 0.001$), AG − C ($p < 0.001$) and AG + D ($p < 0.001$) rats.

Water intakes and AG dosages were also shown in Table I.

### Agonist-induced Contractions

The contractile responses of aortic strips from all groups are shown in Tables II and III. There were no significant differences in contractile responses, either $pD_2$ or $E_{max.}$ values, of aortic

TABLE I Some characteristics of all experimental groups (mean ± SE)

| Groups $n = 10$ | Water intake (ml/day) | AG dosage (mg/kg/day) | Body weights (g) | | | Blood glucose (mg/dl) | | |
|---|---|---|---|---|---|---|---|---|
| | | | Basal | 3. day | 8. week | Basal | 3. day | 8. week |
| Normal Control (NC) | 44 ± 1 | – | 306 ± 13 | 307 ± 13 | 331 ± 14 | 111 ± 3 | 124 ± 5 | 124 ± 3 |
| Diabet Control (DC) | 155 ± 3[1,2] | – | 224 ± 7 | 239 ± 6 | 205 ± 7[5] | 99 ± 2 | 313 ± 52[6,7] | 583 ± 8[8,12–14] |
| AMNG-Control (AG – C) | 41 ± 1 | 79 ± 3 | 313 ± 9 | 315 ± 11 | 315 ± 14 | 108 ± 4 | 128 ± 4 | 135 ± 5 |
| AMNG-treated diabet (AG + D) | 150 ± 3[3,4] | 95 ± 5 | 229 ± 15 | 226 ± 15 | 253 ± 6 | 110 ± 4 | 285 ± 48[9,10] | 480 ± 14[11,15,16] |

[1]DC *vs.* NC $p < 0.001$; [2]DC *vs.* AG – C $p < 0.001$; [3]AG + D *vs.* NC $p < 0.001$; [4]AG + D *vs.* AG – C $p < 0.001$; [5]8.week *vs.* 3. day $p < 0.01$; [6]3.day *vs.* basal $p < 0.001$; [7]3.day *vs.* 8. week $p < 0.001$; [8]8.week *vs.* basal $p < 0.001$; [9]3.day *vs.* basal $p < 0.001$; [10]3.day *vs.* 8.week $p < 0.001$; [11]8.week *vs.* basal $p < 0.001$; [12]DC *vs.* NC $p < 0.001$; [13]DC *vs.* AG – KC $p < 0.001$; [14]DC *vs.* AG + D $p < 0.001$; [15]AG + D *vs.* NC $p < 0.001$; [16]AG + D *vs.* AG – C $p < 0.001$.

TABLE II $pD_2$ values in aortas from AMNG-treated and untreated control and diabetic rats

| | $pD_2$ | | | |
|---|---|---|---|---|
| Groups $n = 10$ | NA | Ach | SNP | KCl |
| Normal Control (NC) | 7.89 ± 0.20 | 6.53 ± 0.27 | 8.50 ± 0.26 | 1.52 ± 0.02 |
| Diabet Control (DC) | 7.29 ± 0.46 | 5.69 ± 0.23 | 7.89 ± 0.21 | 1.63 ± 0.07 |
| AMNG-Control (AG − C) | 7.15 ± 0.18 | 5.93 ± 0.15 | 7.84 ± 0.23 | 1.44 ± 0.03 |
| AMNG-treated diabet (AG + D) | 8.27 ± 0.28 | 6.33 ± 0.22 | 8.22 ± 0.17 | 1.60 ± 0.04 |

TABLE III Maximum contractions to NA and KCl and maximum relaxations to Ach and SNP in diabetic and control rats

| | $E_{max.}$ (mg tension/mg wet weight) | | % Max. relaxation |
|---|---|---|---|
| Groups $n = 10$ | NA | KCl | Ach |
| Normal Control (NC) | 467 ± 29 | 237 ± 8 | 78 ± 4 |
| Diabet Control (DC) | 425 ± 38 | 250 ± 20 | $23 \pm 5^{1,3}$ |
| AMNG-Control (AG − C) | 356 ± 15 | 221 ± 10 | $47 \pm 8^{2}$ |
| AMNG-treated diabet (AG + D) | 342 ± 24 | 225 ± 5 | 58 ± 8 |

[1]DC *vs.* NC $p < 0.001$; [2]NC *vs.* AG − C $p < 0.01$; [3]DC *vs.* AG + D $p < 0.01$.

TABLE IV The fast, slow and total components of contraction induced by noradrenaline in all the experimental groups

| Groups $n = 10$ | Total | Fast | Slow |
|---|---|---|---|
| Normal Control (NC) | 450 ± 34 | 275 ± 20 | 175 ± 16 |
| Diabet Control (DC) | 397 ± 54 | 232 ± 31 | 165 ± 25 |
| AMNG-Control (AG − C) | 370 ± 22 | 233 ± 18 | 137 ± 11 |
| AMNG-treated diabet (AG + D) | 333 ± 26 | 216 ± 14 | 117 ± 13 |

strips to NA and KCl. In addition, as shown in Table IV, either fast or slow components of responses to noradrenaline were not significantly different among all the experimental groups.

### Agonist-induced Relaxations

The acetylcholine-mediated relaxation ($pD_2$ and %Max. Relax.) of aortic strips precontracted with NA is shown in Tables II and III. Either the diabetic state or AMNG treatment had no effect upon the $pD_2$ pattern of relaxations in all groups. In contrast, the maximum relaxations generated by aortic strips from diabetic rats to Ach were markedly smaller ($p < 0.001$) than those generated by corresponding control tissues (NC) from age-matched rats. Treatment of diabetic animals with AMNG prevented the diabetes-induced depression of relaxations to Ach (DC *vs.* AG + D, $p < 0.01$).

Table II illustrates the $pD_2$ values for SNP from all groups. It was observed that there were no differences in the $pD_2$ values among all the experimental groups.

### DISCUSSION

Vascular deterioration is one of the complicating features of human and experimental diabetes and hyperglycaemia is the primary cause of diabetic micro and macro vascular complications.[1,2] There are several variables which should perhaps be considered when comparing results: strain of rat employed, drug employed to induce diabetes, duration of diabetes, age of animals, presence or absence of endothelium, and the method used to calculate maximum response.[10–14, 19, 20]

It has been shown that hyperglycaemia which is one of the most important markers of diabetes, caused tissue damage with several mechanisms, including advanced glycation end product (AGE) formation, increased polyol pathway flux, apoptosis and reactive oxygen species (ROS) formation.[3, 8, 9, 21] Endothelial dysfunction and impaired endothelium-dependent relaxations have been consistently demonstrated with the histologic and experimental studies in animal models of diabetes mellitus.[19, 22–27] The present results also demonstrated that in aortas from STZ-diabetic rats, the endothelium-dependent relaxant responses to Ach ($25 \pm 5\%$) was significantly decreased compared with NC ($74 \pm 4\%$, $p < 0.001$) rats. Relaxation in the AMNG treated-diabetic group (AG + D) was significantly greater than that seen in the diabetic control (DC) group and % Max. Relax. values returned to near-control values (NC) ($58 \pm 8\%$). Our results are in agreement with the majority of previous studies[22, 23–27] although others have reported no difference in vascular responsiveness to Ach in diabetic rats.[28, 29] The endothelium-dependent relaxant responses to agents such as acetylcholine are largely due to release of endothelium derived relaxing factor (EDRF).[30] EDRF is now considered to be identical to nitric oxide which is a key transducer of the vasodilator signalling.[30, 31] Impaired endothelium-dependent relaxation in STZ-induced diabetic rat might be due to increased blood glucose level, decreased blood insulin level, decreased influx of $Ca^{2+}$ into endothelium or decreased release of $Ca^{2+}$ from its storage sites, a decreased content or inactivation of NO syntase and decreased diffusion of NO into the smooth muscle.[58] In addition, diabetes is believed to cause endothelial damage *via* oxidative stress which induces ROS and lipid peroxidation and a diabetes-induced functional change in vascular endothelial cells could be a key event in the development of the altered endothelium-dependent vasoreactivity.[32, 33]

AMNG used in this study is a potential therapeutic agent for preventing the generation of advanced glycation end products in diabetes mellitus.[34] Although Crinjns *et al.* (1998) have reported no beneficial effect of AMNG-treatment,[45] Bucala *et al.* (1991) previously demonstrated that acceleration of the advanced glycosylation process *in vivo* results in a time-dependent impairment in endothelium-dependent relaxation and inhibition of advanced glycosylation with aminoguanidine prevents nitric oxide quenching, and ameliorates the vasodilatory impairment.[35] These results agree with our findings. In addition, a number have suggested that AGEs decrease NO and cGMP levels and vascular mechanical properties and AMNG-treatment ameliorates this disturbances.[35, 36, 38] On the other hand, AMNG could increase the release of vasodilator substances such as prostacylin,[37] and inhibit ROS formation[17] and oxidant-induced apoptosis.[18] An alternative explanation for beneficial effect of AMNG is its role on the polyol pathway. It has been shown that polyol pathway is related with the deficit for endothelium-dependent relaxation and aldose reductase inhibitors can prevent this deficit in aorta from STZ-diabetic rats[13] and Kumari *et al.*, have recently demonstrated that AMNG is an aldose reductase inhibitor.[39, 40]

The majority of previous studies showed that the responsiveness to the endothelium-independent vasodilator, SNP, is not impaired in diabetics *versus* control[33, 43–46] whilst some has demonstrated an impaired response.[41, 42] This study have also showed an unchanged sensitivity and a maximum relaxation to SNP in diabetics. Our results agree with the majority of previous studies.[33, 43–46]

The contractile response of the rat aorta to noradrenaline, a nonselective alpha agonist, is biphasic, consisting of a fast and a slow component.[47] The fast component of the contraction induced by noradrenaline is a consequence of activation of alpha-1 adrenoceptors and has been demonstrated to be due to mobilisation of intracellular calcium whereas the slow component reflects activation of alpha-2 adrenoceptors and is directly dependent upon an influx of extracellular calcium.[48,49] Despite the controversy concerning the effects of diabetes on the maximal response, sensitivity, fast and slow components of response to the alpha-adrenoceptor agonist, such as noradrenaline,[49–52] most studies agree that agonist potency is not altered by diabetes.[20,53–55] The present study has also demonstrated an unchanged sensitivity, a maximum contraction and its components to noradrenaline in diabetes. Our results are in agreement with some of the previous studies.[20,53,54,55] The discrepancy between our results and those of other groups may be due to the different experiment protocols. In the present study, there were also no significant differences between the diabetic tissues and control tissues in the responsiveness to KCl. However, although some authors found conflicting results,[56,57] the present results agree with those of other groups.[28,54]

In conclusion, our study demonstrates that the chronic AMNG-treatment reversed the diminished vascular relaxation. However, its exact mechanism of action remain unclear and requires further investigation.

### *Acknowledgement*

This work was supported by the Research Fund of University of Istanbul, Project number: 1116/010598.

### *References*

[1] The Diabetes Control and Complications Trial Research Group. (1993). The effect of intensive treatment of diabetes on the development and progression of long-term complications in insulin-dependent diabetes mellitus. *N. Engl. J. Med.*, **329**, 977–986.

[2] Semenkovich, C. F. and Heinecke, J. N. (1997). The mystery of diabetes and atherosclerosis: time for a new plot. *Diabetes*, **46**, 327–334.

[3] DeRubertis, F. R. and Craven, P. A. (1994). Activation protein kinase C in glomerular cells in diabetes: mechanisms and potential links to the pathogenesis of diabetic glomerulopathy. *Diabetes*, **43**, 1–8.

[4] King, G. L., Shiba, T., Oliver, J., Inoguchi, T. and Bursell, S. E. (1994). Cellular and molecular abnormalities in the vascular endothelium of diabetes mellitus. *Ann. Rev. Med.*, **45**, 179–188.

[5] Williamson, J. R., Chang, K., Frangos, M., Hasan, K. S., Ido, Y., Kawamura, T., Nyengoard, J. R., van den Ender, M., Kilo, C. and Tilton, R. G. (1993). Hyperglycaemic pseudohypoxia and diabetic complications. *Diabetes*, **42**, 801–813.

[6] Bucala, R., Makita, Z., Koschinsky, T., Cerami, A. and Ulassara, H. (1993). Lipid advanced glycosylation: pathway for lipid oxidation *in vivo*. *Proc. Watl, Acad. Sci. USA*, **90**, 6434–6438.

[7] Vlassara, H., Bucala, R. and Striker, L. (1994). Pathogenic effects of AGE's: biochemical, biological and clinical implications for diabetes and ageing. *Lab. Invest.*, **70**, 138–151.

[8] Brownlee, M., Cerami, A. and Vlassara, H. (1998). Advanced glycosylation and products in tissue and the biochemical basis of complications. *N. Engl. J. Med.*, **318**, 1315–1321.

[9] Hammes, H. P., Federoff, H. J. and Brownlee, M. (1995). Nerve growth factor prevents neuroretinal programmed cell death and capillary pathology in experimental diabetes. *Mol. Med.*, **1**, 527–534.

[10] Agrawal, D. K. and Mc Weill, J. H. (1987). Vascular responses to agonist in rat mesenteric artery from diabetic rats. *Can. J. Physiol. Pharmacol.*, **65**, 1484–90.

[11] Takiguchi, Y., Saton, N., Hashimoto, H. and Nakashima, M. (1989). Reversal effect of thyroxine on altered vascular reactivity in diabetic rats. *J. Cardiovasc. Pharmacol.*, **13**, 520–524.

[12] White, R. F. and Carrier, G. O. (1990). Vascular contraction induced by activation of membrane calcium ion channels is enhanced in streptozotocin-diabetes. *J. Pharmacol. Exp. Ther.*, **253**, 1057–1062.

[13] Cameron, N. E. and Catter, M. A. (1992). Impaired contraction and relaxation in aorta from streptozotocin diabetic rats: role of polyol pathway. *Diabetologia*, **35**, 1011–1019.

[14] Taylor, P. D., Mc Carthy, A. L., Thmas, C. R. and Poston, L. (1992). Endothelium-dependent relaxation and noradrenaline sensitivity in mesenteric resistance arteries of streptozotocin-induced diabetic rats. *Br. J. Pharmacol.*, **107**, 393–9.

[15] Yagihashi, S., Kamijo, M., Baba, M., Yagihashi, N. and Nagai, K. (1992). Effect of aminoguanidine on functional and structural abnormalities in peripheral nerve of STZ-induced diabetic rats. *Diabetes*, **41**, 47–52.

[16] Bucala, R., Cerami, A. and Vlassara, H. (1995). Advanced glycosylation end product in diabetic complications. *Diabetes Rev.*, **3**, 258–268.

[17] Griffiths, M. J., Messent, M., Mac Allister, R. J. and Evans, T. W. (1993). Aminoguanidine selectively inhibits inducible nitric oxide synthase. *Br. J. Pharmacol.*, **110**(3), 963–968.

[18] Giardino, I. F., Arman, K., Hatechell, D. L. and Browniee, M. (1998). Aminoguanidine inhibits reactive oxygen species formation, lipid peroxidation and oxidant-induced apoptosis. *Diabetes*, **47**(7), 1114–1120.

[19] Kamata, K., Miyata, N. and Kasuya, Y. (1989). Impairment of endothelium-dependent relaxation and changes in levels of cyclic GMP in aorta from streptozotocin induced diabetes rats. *Br. J. Pharmacol.*, **97**(2), 614–618.

[20] Heygate, K. M., Lawrence, I. G., Bennett, M. A. and Thurston, H. (1995). Impaired endothelium-dependent relaxation in isolated resistance arteries of spontaneously diabetic rats. *Br. J. Pharmacol.*, **116**(8), 3251–3259.

[21] Dieper, G. M. and Gross, G. J. (1989). Selective impairment of endothelium-dependent relaxation by oxygen-derived free radicals: distinction between receptor *versus* non receptor mediator. *Blood Vessels*, **26**, 44–47.

[22] Agrobast, B. W., Berry, D. L. and Newell, C. L. (1984). Injury of arterial endothelial cells in diabetic. Sucrose fed and aged rats. *Atherosclerosis*, **51**, 31–45.

[23] Hattori, Y., Kawasaki, H., Abe, K. and Kanno, M. (1991). Superoxide dismutase recovers altered endothelium dependent relaxation in diabetic rat aorta. *Am. J. Physiol.*, **261**, H1086–H1094.

[24] Öztürk, M., Tunçdemir, M., Bolkent, S., Kaya, S., Yilmazer, S., Akkan, A. G., Özyazgan, S., Senses, V. and Özüner, Z. (1997). An ultrastructural study on the effects of AICA-riboside treatment on aortic alteration in neonatal STZ-induced diabetic rats. *13th. National Electron Microscopy Congress Proceedings, Ankara*, pp. 335–340.

[25] Oyama, Y., Kawasaki, H., Hattori, Y. and Kanno, M. (1986). Attenuation of endothelium-dependent relaxation in aorta from diabetic rats. *Eur. J. Pharmacol.*, **131**, 75–78.

[26] Durante, W., Sen, A. K. and Sunahara, F. A. (1988). Impairment of endothelium-dependent relaxation in aorta from spontaneously diabetic rats. *Br. J. Pharmacol.*, **94**, 463–468.

[27] Tesfamariam, B. and Cohen, R. A. (1992). Free radicals mediate endothelial cell dysfunction caused by elevated glucose. *Am. J. Physiol.*, **263**, H321–H326.

[28] Fiol de Cuneo, M., Ruiz, R. D., Lacnara, J. L. and Santillan de Tores, R. (1988). Contractility and pharmacological reactivity of isolated vascular smooth muscle from diabetic rats. *Pharmacology*, **36**(4), 228–237.

[29] Agrawal, D. K., Bhimji, S. and McNeil, J. H. (1987b). Effect of chronic experimental diabetes on vascular smooth muscle function in rabbit carotid artery. *J. Cardiovasc. Pharmacol.*, **9**(5), 584–593.

[30] Furchgott, R. F. and Zawadzki, J. V. (1980). The obligatory role of endothelial cells in the relaxation of smooth muscle cells by acetylcholine. *Nature*, **288**, 373–376.

[31] Palmer, R. M. J., Ferrige, A. G. and Moncada, S. (1987). Nitric oxide account the biological activity of endothelium-derived relaxing factors. *Nature*, **327**, 524–526.

[32] Ceriello, A., Quatraro, A. and Guigliano, D. (1979). Diabetes Mellitus and hypertension: the possible role of hyperglycaemia through oxidative stress. *Diabetologia*, **17**, 371–377.

[33] Young, I. S., Tate, S., Lightbody, J. H., Mcmaster, D. and Trimble, E. R. (1995). The effect of desferoxamine and ascorbate on oxidative stress in the streptozotocin diabetic rats. *Free Radic. Biol. Med.*, **18**, 833–840.

[34] Brownlee, M., Vlassara, H., Kooney, A., Ulrich, P. and Cerami, A. (1986). Aminoguanidine prevents diabetes-induced arterial wall protein cross-linking. *Science* (*Wash. DC*), **232**, 1629–1632.

[35] Bucala, R., Tracey, K. J., Cerami, A. (1991). Advanced glycosylation products quench nitric oxide and mediate defective endothelium-dependent vasodilatation in experimental diabetes. *J. Clin. Ýnvest.*, **87**, 432–438.

[36] Yamagishi, S., Fujimori, H., Yonekura, H., Yamamoto, Y. and Yamamoto, H. (1998). Advanced glycation and products inhibit prostacyclin production and induce plasminogen activator inhibitor-1 in human microvascular endothelial cells. *Diabetologia*, **41**, 1425–1441.

[37] Dewhurst, M., Stevens, E. J., Karasu, C. and Tomlinson, D. R. (1994). Effects of aminoguanidine treatment on sciatic nerve laser Doppler flux and prostacyclin release from vasculer tissues and sciatic nerve of control and streptozotocin diabetic rats. *Br. J. Pharmacol.*, **112**, 373.

[38] Huijberts, M. S. P., Wolffenbuttel, B. H. R., Boudier, H. A. J. S., Crijns, F. R. L., Kruseman, A. C. N., Poitevin, P. and Levy, B. I. (1993). Aminoguanidine treatment increases elasticity and decreases fluid filtration of large arteries from diabetic rats. *J. Clin. Invest.*, **92**, 1407–1411.

[39] Kumari, K., Uman, S., Bansal, V., Sahib, M. K. (1991a). Mono aminoguanidine inhibits aldose reductase. *Biochem. Pharmacol.*, **15**; **41**(10), 1527–1528.

[40] Kumari, K., Uman, S., Bansal, V. and Sahib, M. K. (1991b). Inhibition of diabetes-associated complications by nucleophilic compounds. *Diabetes*, **40**(8), 1079–84.

[41] Taylor, P. D., Graves, J. E. and Poston, L. (1995). Selective impairment of acetylcholine-mediated endothelium-dependent relaxation in isolated resistance arteries of the streptozotocin-induced diabetic-rat. *Clin. Sci.*, **88**, 519–524.

[42] Kiff, R. J., Gardiner, S. M., Comptom, A. M. and Bennett, T. (1991). Selective impairment of hindquarter vasodilator responses to bradykinin in conscious Wistar rats with STZ-induced diabetes mellitus. *Br. J. Pharmacol.*, **103**, 1357–1362.

[43] Hattori, Y., Kawasaki, H., Kamno, M., Gando, S. and Fukao, M. (1994). Attenuated contractile response of diabetic rat aorta to caffeine but not to adrenaline in $Ca^{++}$-free medium. *Eur. J. Pharmacol.*, **256**(2), 215–219.

[44] Rembold, C. M. (1992). Regulation of contraction and relaxation in arterial smooth muscle. *Hypertension*, **20**, 129–137.

[45] Crijns, F. R. L., Boudier, S. H. A. J. and Wolffenbuttel, B. H. R. (1998). Arteriolar reactivity in conscious diabetic rats: Influence of aminoguanidine treatment. *Diabetes*, **47**, 918–923.

[46] Archibald, V., Cotter, M. A., Keegan, A. and Cameron, N. E. (1996). Contraction and relaxation of aortas from diabetic rats: Effects of chronic anti-oxidant and aminoguanidine treatment. *Naunyn-Schmiedebergs-Arch-Pharmacol.*, **353**(5), 584–91.

[47] Bohr, D. F. (1963). Vascular smooth muscle. Dual effects of calcium. *Science*, **139**, 597–599.

[48] Scarborough, N. L. and Carrier, G. O. (1984a). Nifedipine and alpha adrenoceptors in rat aorta: I. Role of extracellular calcium in alpha-1 and alpha-2

adrenoceptor-mediated contraction. *J. Pharmacol. Exp. Ther.*, **231**, 597–602.

[49] Scarborough, N. L. and Carrier, G. O. (1984b). Nifedipine and alpha adrenoceptors in rat aorta: II. Role of extracellular calcium in enhanced alpha-2 adrenoceptor-mediated contraction. *J. Pharmacol. Exp. Ther.*, **231**, 603–609.

[50] Mc Leod, K. M. and Mc Neill, J. H. (1982). Alpha adrenoceptor mediated responses in aorta from three month streptozotocin-diabetic rats. *Proc. West. Pharmacol. Soc.*, **25**, 245–247.

[51] Sullivan, S. and Spark, H. V. (1979). Diminished contractile response of aortas from diabetic rabbits. *Am. J. Physiol.*, **236**, 301–306.

[52] Fortes, Z. B., Leme, J. G. and Scivoletto, R. (1983). Vascular reactivity in diabetes mellitus. Role of the endothelial cell. *Br. J. Pharmacol.*, **79**, 771–781.

[53] Özyazgan, S., Senses, V., Ince, E., Sultuybek, G., Utkan, T. and Akkan, A. G. (1998). The effect of succinic acid monomethyl ester (SAM) on the responses of isolated thoracic aorta in streptozotocin-diabetic rats. *Pharmacol. Res.*, **38**(1), 73–79.

[54] Mulherm, M. and Docherty, R. J. (1989). Effect of experimental diabetes on the responsiveness of rat aorta. *Br. J. Pharmacol.*, **97**, 1007–1012.

[55] Harris, K. H. and Mc Leod, K. M. (1988). Influence of endothelium on contractile responses of arteries from diabetic rats. *Eur. J. Pharmacol.*, **153**(1), 55–6.

[56] Owen, M. P. and Carrier, G. O. (1979). Alteration in vascular smooth muscle sensitivity to vasoconstrictor agents by streptozotocin-induced diabetes. *Proc. West Pharmacol. Soc.*, **22**, 363–366.

[57] Ramanadham, S., Lyness, W. H. and Tenner, T. E. (1984). Alteration in aortic and tail artery reactivity to agonists after streptozotocin treatment. *Can. J. Physiol. Pharmacol.*, **62**(4), 418–23.

[58] Kamata, K. and Nakojima, M. (1998). $Ca^2$ mobilisation in the aortic endothelium in streptozotocin-induced diabetic and cholesterol-fed mice. *Br. J. Pharmacol.*, **123**, 1509–1516.

